# Efficacy and Safety of Manual Partial Red Cell Exchange in the Management of Severe Complications of Sickle Cell Disease in a Developing Country

**DOI:** 10.1155/2017/3518402

**Published:** 2017-05-11

**Authors:** B. F. Faye, D. Sow, M. Seck, N. Dieng, S. A. Toure, M. Gadji, A. B. Senghor, Y. B. Gueye, D. Sy, A. Sall, T. N. Dieye, A. O. Toure, S. Diop

**Affiliations:** ^1^Hematology, Cheikh Anta Diop University, BP 5005, Dakar, Senegal; ^2^Centre National de Transfusion Sanguine, BP 5002, Dakar, Senegal

## Abstract

**Introduction:**

The realization of red cell exchange (RCE) in Africa faces the lack of blood, transfusion safety, and equipment. We evaluated its efficacy and safety in severe complications of sickle cell disease.

**Patients and Method:**

Manual partial RCE was performed among sickle cell patients who had severe complications. Efficacy was evaluated by clinical evolution, blood count, and electrophoresis of hemoglobin. Safety was evaluated on adverse effects, infections, and alloimmunization.

**Results:**

We performed 166 partial RCE among 44 patients including 41 homozygous (SS) and 2 heterozygous composites SC and 1 S/*β*0-thalassemia. The mean age was 27.9 years. The sex ratio was 1.58. The regression of symptoms was complete in 100% of persistent vasoocclusive crisis and acute chest syndrome, 56.7% of intermittent priapism, and 30% of stroke. It was partial in 100% of leg ulcers and null in acute priapism. The mean variations of hemoglobin and hematocrit rate after one procedure were, respectively, +1.4 g/dL and +4.4%. That of hemoglobin S after 2 consecutive RCE was −60%. Neither alloimmunization nor viral seroconversion was observed.

**Conclusion:**

This work shows the feasibility of manual partial RCE in a low-resource setting and its efficacy and safety during complications of SCD outside of acute priapism.

## 1. Introduction

Transfusion therapy is the cornerstone of the management of sickle cell disease (SCD) [1]. It reduces significantly the morbidity and mortality [1–3]. In the study “*stroke with transfusions changing to hydroxyurea (SWiTCH)*” chronic transfusion proved to be the best preventive option of stroke among sickle cell patients who had a cerebral vasculopathy [4, 5]. The National Heart Lung and Blood Institute (NHLBI) guidelines, 2014, strongly recommend transfusion, in particular red cell exchange (RCE), in several other indications such as acute chest syndrome (ACS), stroke, hepatic sequestration, and multisystem organ failure [6]. However transfusion increases blood viscosity particularly in patients whose rate of hemoglobin is higher than 10 g/dL. Thus it can participate in the occurrence of vasoocclusive complications [1, 2, 7]. Also, chronic transfusion therapy exposes to the risk of iron overload [1, 7] while no iron chelator treatment is available now in our country. Partial RCE consists on replacing a part of the blood of sickle cell patients by another from donors who are free of SCD [8]. It reduces the hemoglobin S (HbS) rate, brings normal hemoglobin without increasing the hemoglobin rate where hyperviscosity is a risk, and decreases iron overload [2, 8]. The method can be automated using cytapheresis or manual based on the realization of a bleeding followed by a transfusion of red blood cells [2]. The automated RCE has already proved its efficacy and safety in the developed countries [2, 3, 9]. One of the major limits for its use is the high cost of its equipment which makes it unavailable in centers with limited resources [9, 10]. In the comparative study of Koehl et al. the cost of automated RCE was 74 times higher than the manual method relating to equipment cost [11]. Manual method however has the advantage of being more accessible and not expensive and requires few tools [10]. In our clinical unit, the cost of one session of partial RCE is only 11.4 euros (7500 frs CFA). Its procedure differs from one center to another and depends largely on local resources [9]. The aim of this study was to evaluate the feasibility, efficacy, and safety of an easy protocol of manual partial RCE in adult sickle cell patients with severe complications.

## 2. Patients and Method

We conducted a prospective study from 11/01/2012 to 02/28/2015 (28 months).

### 2.1. Patients

All types of SCD in patients older than 16 years with severe complications were included. RCE was performed in the first time of the management in patients with stroke, severe ACS, and acute priapism. In those with persistent vasoocclusive crisis (PVOC), recurrent priapism, or leg ulcer, it was done after failure of the medical treatment.

Patients with a hemoglobin rate lower than 6 g/dL and those with cardiac failure or severe renal deficiency were not included.

### 2.2. Method

#### 2.2.1. Setting

The Clinical Hematology Department is a public center located in the National Blood Transfusion Center, next to the blood donation department and his medical laboratory. It includes a medical consultation unit where about 20 patients are checked daily and an inpatient unit of 20 beds. It is the national reference center for adult SCD, malignant hemopathies, and hemophilia. It also welcomes students of the Faculty of Medicine and Pharmacy during their training. Pre- and posttransfusion tests are performed in the medical laboratory of the National Blood Transfusion Center.

#### 2.2.2. Pretherapeutic Assessment

It included a clinical examination, blood count, blood group determination, electrophoresis of hemoglobin, serology of HIV, hepatitis B, hepatitis C, and syphilis.

#### 2.2.3. Characteristics of the Red Cell Units

The red cell units used had approximately a volume of 250 mL, an hematocrit rate about 60%, and an hemoglobin content greater than 45 g. They were preserved in citrate, phosphate, dextrose, and adenine (CPDA). All the units underwent the following tests: ABO and Rhesus group, serology of HIV, hepatitis B, hepatitis C, and syphilis which were negatives, and the sickling-test which was negative too indicating the absence of HbS.

#### 2.2.4. Partial RCE Procedure

All procedures were performed on peripheral venous of the upper limbs by nurses who had been trained in partial manual RCE by the medical team. Each procedure was performed by one nurse. The procedure began with a bleeding in free flow in a blood bag. Its total volume was according to the baseline hemoglobin rate. It was 250 mL when the hemoglobin rate was between 6 and 8 g/dL; 500 mL, 750 mL, and 1000 mL, respectively, for 8.1–10 g/dL, 10.1–12 g/dL, and higher than 12 g/L. It was followed by an intravenous hydration using isotonic saline. Its volume was equal to the bleeding one in order to prevent hypovolemia. In patients whose total volume of the bleeding was 750 mL or 1000 mL, we removed at first 500 mL of blood, gave 500 mL saline, and then removed 250 mL or 500 mL, respectively, to prevent hypovolemia. Then a transfusion of 2 units of red blood cell was done. Finally a last saline hydration with the same volume than the transfusion was carried out in order to prevent hyperviscosity. When after RCE, the hematocrit was higher than 40% or the hemoglobin rate higher than 12 g/dL, an additional bleeding of 500 mL was carried out in order to return below these limits. One procedure of RCE was performed for each episode of recurrent priapism and PVOC. Two consecutive RCE were done at the day of the admission for stoke, severe ACS, and acute priapism. For leg ulcers, one procedure was performed every 4 weeks.

#### 2.2.5. The Monitoring for Adverse Transfusion Reactions

We monitored the blood pressure, pulse, respiratory rate, temperature, oxygen saturation, and consciousness before RCE, every 30 min during the procedure and at the end. At least twice daily after RCE, clinical examination was done for evaluate the evolution of symptoms and screen adverse events. Additional tests depended on the type of reactions suspected.

#### 2.2.6. Evaluation of Efficacy and Safety

The main symptoms evaluated were bone pain in PVOC; chest pain, dyspnea, and oxygen saturation in severe ACS; paralysis, alteration of consciousness, and convulsions in stroke; painful erection in priapism; size reduction in leg ulcers. The regression of symptoms was considered as complete when they disappeared within 4 hours after RCE for acute priapism, 1 day for recurrent priapism, 3 days for PVOC, severe ACS, and stroke, and 1 month for leg ulcer. A blood count was done after every RCE and an electrophoresis of hemoglobin was done after two consecutive RCE. Red cell antibodies screening was performed by the gel card method of the Biorad kit. Serology of HIV, hepatitis B, hepatitis C, and syphilis were tested at the end of the study.

#### 2.2.7. Statistical Study

Data analysis was done using SPSS software version 18. Descriptive study was conducted by calculating frequencies and proportions for qualitative variables. For quantitative data, we calculated the averages with their 95% confidence intervals.

## 3. Results

A total of 166 partial RCE was performed in 44 sickle cell patients including 41 homozygous (SS) and two heterozygous composites SC and one S/*β*0-thalassemia. They were from a cohort of 1120 patients (3.9%). The sex ratio was 1.58; the mean age was 27.9 years [95% IC: 25.8–30].

### 3.1. Indications

The main indications were recurrent priapism (36.1%), PVOC (24%), and chronic leg ulcer (18%) (Table 1).

### 3.2. RCE Parameters

The average duration of RCE was 170 minutes [95% IC: 167–175] per procedure. The average volume of bleeding was 475 mL [95% IC: 439.5–510.5]. That of the transfused blood was 556.6 mL [95% IC: 540.4–572.8] corresponding to 2 red cell units. The total of red blood cell units was 332 with an average of 7.5 units per patient [95% IC: 5.8–10].

### 3.3. Efficacy of RCE

Regression of symptoms was complete in 100% of cases of PVOC and severe ACS, 56.7% of recurrent priapism, and 30% of stroke. It was partial in 100% of leg ulcers, 70% of stroke, and 43.3% of recurrent priapism. Zero percent (0%) of acute priapism cases had obtained regression; thus they were transferred to the urological emergencies (Table 1). The mean variations of the hemoglobin rate and hematocrit after each procedure were, respectively, +1.4 g/dL and +4.4%. That of HbS after 2 consecutive RCE was −60% (Table 2).

### 3.4. Safety of RCE

There were no difficulties of venous access which limited the performing or continuation of RCE. Minor adverse events had occurred in 6 cases (3.6%) such as dizziness, headaches, fever, urticaria (1 case for each of them), and itching (2 cases). Neither alloimmunization nor seroconversion to the HIV, HBV, HCV, and syphilis was observed.

## 4. Discussion

This work shows that given proper training, despite a low-resource setting, manual partial RCE can be safely and successfully performed in the management of several complications of SCD outside the acute priapism. It allows a significant reduction of the hemoglobin S (HbS) rate and brings normal hemoglobin without increasing the hemoglobin rate where hyperviscosity is a risk. This efficacy was variable according to indications. Several authors found variation of the efficacy of partial RCE according to the methods used and the indications [2, 8, 12, 13].

In the cases of PVOC and recurrent priapism, one procedure was performed for each episode because it allowed a favorable evolution. So the variation of HbS rate was not evaluated. In these indications, the clinical evolution is the decisive parameter for the assessment of efficacy. Correlation between the rate of HbS and clinical improvement is not perfect [9]. Two consecutive RCE was done in the vital emergency situations such as severe ACS and stroke to exchange large volumes of blood in order to obtain a significant reduction of HbS level [9]. They were done in two times to prevent adverse events related to a large volume of bleeding. In these cases erythrocytapheresis would be more suitable because it could be performed safely under isovolemia [2, 9]. The efficacy of RCE was less remarkable in stroke (only 30% of complete clinical regression) although HbS had reached the recommended rate of 30% [14]. This could be related to the severity of the cerebral injuries before treatment. Regarding the healing of leg ulcers, it was only partial in 100% of the cases. Minniti et al. found that there is no controlled data that shows the efficacy of chronic transfusion in the healing of chronic leg ulcers in SCD likely due to their multifactorial origin [15].

As for the acute priapism, RCE failed in 100% of the cases. With respect to this outcome, we acknowledge that transfusion is not effective for the treatment of acute priapism according to the 2014 NIH guidelines [6]. The interest of the RCE in the management of the priapism remains controversial [14]. In a literature review conducted by Kato [16], he found that RCE and other drug treatments were not efficient in this indication and delayed the urologic management. However Ballas and Lyon had obtained clinic regression by cytapheresis by maintaining an HbS rate lower than 30% [17].

Concerning the biological response, the average rate of hemoglobin after one RCE (10.3 g/dL) and that of HbS after two consecutive RCE (24.8%) were according to recommendations. The suggested goal is a hemoglobin rate close to but not greater than 10 g/dL and the HbS rate lower than 30% in stroke and lower than 50% in other complications [2, 9, 14].

About safety, a low rate of minor acute adverse events (3,6%) had occurred. Indeed during RCE, acute complications are usually rare and transitory [12, 18].

No infection by HIV, HBV, and HCV was observed. In Senegal the safety against infections related to transfusion has improved thanks to the efficient strategies in medical selection of blood donors [19, 20] and better techniques in the screening of infectious diseases [21]. Despite these advances, the risk of infections related transfusion remains high in Senegal and in Africa in general. So it limits the indications of chronic transfusion in these countries [7, 19, 21–24].

No case of alloimmunization was observed either. It could partly be due to a better homogeneity between the blood group antigens in donors and patients. In a systematic review and meta-analysis done by Ngoma et al. about red blood cell alloimmunization in transfused patients in sub-Saharan Africa, overall proportions of alloimmunization were 6.7 (95% CI: 5.7–7.8) per 100 transfused patients [25]. In Europe where black sickle cell patients usually receive blood from white donors, the risk of alloimmunization is higher due to a greater antigenic difference [1, 7, 23]. Michot et al. found in their cohort that the prevalence of the alloimmunization was 33% [26]. However, Venkateswaran et al. had shown that when the RCE is done with Rhesus and Kell system matched, it does not increase the risk of allo- or autoimmunization more than simple chronic transfusion despite the exposure to a larger number of red cell units [27].

## 5. Conclusion

This work shows that, given proper training, despite a low-resource setting, manual partial RCE can be safely and successfully performed in the management of several complications of SCD. Its efficacy is variable according to indications. It allows a significant reduction of the hemoglobin S (HbS) rate and brings normal hemoglobin without increasing the hemoglobin rate where hyperviscosity is a risk. However it should not delay the urologic management in acute priapism. A larger study should better evaluate the quality of this treatment and the associated difficulties such as iron overload.

## Figures and Tables

**Table 1:** Indications and clinical evolution of patients after RCE.

Indications	Number of patients (*N* = 44)	Number of RCE per patient	Total number of RCE (*N* = 166)	Regression of symptoms		Mean delay before regression (day)
				Complete (%)	Partial (%)	
Recurrent priapism^*∗*^	12	5	60	56,7	43,3	1
Acute priapism^*∗∗∗*^	6	2	12	0	0	NA
PVOC^*∗*^	8	5	40	100	0	2,4
Leg ulcer^*∗∗*^	6	5	30	0	100	21
Stroke^*∗∗∗*^	10	2	20	30	70	3,2
Severe ACS^*∗∗∗*^	2	2	4	100	0	1,7

^*∗*^Clinical evaluation after one RCE; the other episodes had occurred several months later.

^*∗∗*^Clinical evaluation conducted monthly during 5 months.

^*∗∗∗*^Clinical evaluation conducted after 2 consecutive RCE performed the day of admission.

**Table 2:** Evaluation of the blood count parameters after one RCE and hemoglobin fractions after 2 consecutive RCE performed the day of admission.

Parameters	Values				Mean variation
	Baseline		Final		
	Mean	95% CI	Mean	95% CI	
*Blood count before and after one RCE *(*N* = 166)					
Red cells count (10^12^/L)	3,1	2,9–3,3	3,3	3,1–3,4	+0,2
Hemoglobin rate (g/dL)	8,9	8,6–9,1	10,3	8,5–12,2	+1,4
Hematocrit (%)	26,2	24,7–27,8	30,6	23,3–37,6	+4,4
White cells count (10^9^/L)	11,5	10,7–12,2	11,6	10,4–12,7	+0,1
Platelets count (10^9^/L)	443	414–473	433	401–475	−10
*Fractions of hemoglobin before and after 2 RCE performed the day of admission *(*N* = 12)					
Hemoglobin S (%)	84,8	80,5–89	24,8	20,6–32	−60
Hemoglobin A (%)	0	—	64	58–70	+64
Hemoglobin F (%)	10,2	8,0–12,5	6	3,0–7,5	−4,2
Hemoglobin A2 (%)	3,3	2,9–3,7	2,3	1,9–3,0	−1
